# Proteomic analysis of gametophytic sex expression in the fern *Ceratopteris thalictroides*

**DOI:** 10.1371/journal.pone.0221470

**Published:** 2019-08-19

**Authors:** Xuefei Chen, Zhiyi Chen, Wujie Huang, Huanhuan Fu, Quanxi Wang, Youfang Wang, Jianguo Cao

**Affiliations:** 1 College of Life Science, East China Normal University, Shanghai, China; 2 College of Life Science, Shanghai Normal University, Shanghai, China; Northeast Forestry University, CHINA

## Abstract

*Ceratopteris thalictroides*, a model fern, has two kinds of gametophytes with different sex expression: male and hermaphrodite. Hermaphroditic gametophytes have one or several archegonia beneath the growing point and a few antheridia at the base or margin. Male gametophytes show a spoon-like shape with much longer than the width and produce many antheridia at the margin and surface. The results of chlorophyll fluorescence detection showed that the photochemical efficiency of hermaphrodites was higher than that of males. By using two-dimensional electrophoresis and mass spectrometry, the differentially abundant proteins in hermaphroditic and male gametophytes were identified. A total of 1136 ± 55 protein spots were detected in Coomassie-stained gels of proteins from hermaphroditic gametophytes, and 1130 ± 65 spots were detected in gels of proteins from male gametophytes. After annotation, 33 spots representing differentially abundant proteins were identified. Among these, proteins involved in photosynthesis and chaperone proteins were over-represented in hermaphrodites, whereas several proteins involved in metabolism were increased in male gametophytes in order to maintain their development under relatively nutritionally deficient conditions. Furthermore, the differentially abundant cytoskeletal proteins detected in this study, such as centrin and actin, may be involved in the formation of sexual organs and are directly related to sex expression. These differentially abundant proteins are important for maintaining the development of gametophytes of different sexes in *C*. *thalictroides*.

## Introduction

Sex expression is an important stage in the development of plants. In angiosperms and gymnosperms, the gametophytic phase is very short, and male and female gametophytes are formed in differentiated sporophyte structures [1]. In dioecious species, the sex expression of plants exhibits plasticity that is induced by environmental factors [1, 2]. Environmental resource limitation is considered to be an important reason that dioecious plants develop into male-biased populations [3–5].

In ferns and lycophytes, which have independent living gametophytes sex expression occurs in gametophytes. The gametophytes of homosporous ferns differentiate in response to environmental cues perceived soon after spore germination [2]. This phenomenon is known as environmental sex determination (ESD), and it generally causes individuals in favourable conditions (high light, water and nutrient availability) to develop into females. Whereas individuals in unfavourable conditions (due to limited growth resources, parasites, physical injuries, dry soils and high temperatures) tend to develop into males [6]. For example, high levels of light can modify the sex ratio of *Equisetum* gametophytes to favour females, whereas the presence of excess sugar in culture media can favour males [7, 8]. Limited nutrients supply induced maleness in *Woodwardia*, and a high culture density increased the percentage of males or asexuals in *Osmunda* [9, 10].

*Ceratopteris thalictroides* is a homosporous fern whose spores can develop into male or hermaphroditic gametophytes under the influence of ESD. Studies in a plant from same genus, *C*. *richardii*, have been widely reported. Previous studies have shown that the default sexual development pathway in *C*. *richardii* results in hermaphrodites; subsequently, hermaphrodites secrete antheridiogen, a male-inducing pheromone, into the environment, which causes later germinated spores to develop into males due to the influence of antheridiogen [11–13]. When antheridiogen is removed from the environment, males may transform into hermaphrodites [14]. The sex determination pathway in *C*. *richardii* has been explored, and it has been found that there are two antagonistic major regulators of sex identification, *FEM1* and *TRA*, in the sex-determining pathway [15, 16]. A transcriptome analysis of *C*. *richardii* gametophytes showed that genes involved in epigenetic reprogramming, hormone responses and developmental genes were decreased in male gametophytes induced with antheridiogen [17]. In addition to hormones, nutritional deficiency can also cause spores to grow into male gametophytes. We found that when grown on Knop's medium, a medium free of sucrose and hormones, most *C*. *thalictroides* spores grow into male gametophytes in culture. However, on Murashige and Skoog medium (MS medium), *C*. *thalictroides* spores always grow into hermaphroditic gametophytes. The proteomic approach seems to be the most suitable way to reveal the direct physiological processes involved in plant development [18]. The mechanisms involved in fern development and stress responses have been studied with proteomics approaches [19–21]. Previous studies on *Blechnum spicant* have found that female and male gametophytes have enrichment differences in proteins related to stress or defense, protein biosynthesis, and photosynthesis [22]. However, relatively few studies have been conducted on the differences in growth in male and hermaphroditic gametophytes in *C*. *thalictroides*. Therefore, in the present paper, proteomics was used to analyse differences in morphology, photochemical efficiency, and protein abundance analysis in male and hermaphroditic gametophytes to understand the effects of sex expression on growth and development in *C*. *thalictroides* gametophytes.

## Materials and methods

### Material cultures and morphological observations

*Ceratopteris thalictroides* (L.) Brongn spores were collected from the Botanical Garden of Shanghai Normal University. The spores were grown on MS and modified Knop's solid media in an artificial climate incubator. The incubator conditions consisted of a day period at 25±1°C under normal illumination (approximately 43 μmol m ^-2.^ s ^-1^) with a photoperiod of 18 hours and a night period at 22±1°C. Every seven days, the gametophytes were observed with a Nikon E800 microscope, and the ratio of male to hermaphroditic gametophytes in each dish was recorded.

Different gametophytes were selected under a dissection microscope, fixed in 3% glutaraldehyde for 6 hours, and then incubated in 2% osmium tetroxide solution at room temperature for 2 hours. The samples were dehydrated with an acetone series (30%, 50%, 70%, 90%, and 100%) and finally embedded in Spurr's resin. The embedding block was cut into slices (1 to 2 μm) with a glass cutter and stained with toluidine blue. Observation and micrography were performed with a light microscope.

### Variation of chlorophyll fluorescence in male and hermaphroditic gametophytes

The chlorophyll fluorescence parameters of gametophytes were determined using a Dual2PAM2100 chlorophyll fluorescence analyzer (Walz). Male and hermaphroditic gametophytes were individually wrapped with preservative film. After 2 hours incubation in the dark, the electron transfer quantum efficiency (ФPSⅡ) and the photochemical efficiency (Fv / Fm) were measured under room light. The experiment was repeated 3 times and averaged. Single-factor analysis of variance and multiple comparisons were performed using SPSS 17.0 software.

### Protein extraction

Male and hermaphroditic gametophytes were observed by microscopy to ensure that the harvested gametophytes had greater than 95% purity. After harvesting, the samples were frozen immediately in liquid nitrogen and stored at -80°C until use. Total protein was extracted using a phenol extraction method. Protein powder (3 g) was solubilized in 10 ml of protein extract buffer (0.9 M sucrose, 0.1 M Tris-HCl, pH 8.8, 10 mM EDTA, and 0.4% β-mercaptoethanol) and 10 ml of Tris-saturated phenol. Then, the suspension was shaken for 30 min to completely dissolve the proteins. After centrifugation at 15 000 × g for 10 min at 4°C, the phenolic phase was collected into a new tube. Five millilitres of protein extract buffer and 5 ml of phenol were added to the lower phase, which was again shaken for 30 min and centrifuged as described above. The phenolic phase was consolidated, and 5 volumes of 100 mM ammonium acetate/methanol solution was added. The proteins were precipitated overnight at -20°C. After centrifugation at 20 000 × g for 15 min at 4°C, the supernatant was removed, and the precipitate was rinsed three times in 10 ml of 100 mM ammonium acetate/methanol solution and then twice in 10 ml of 80% acetone. The final precipitate was air-dried and solubilized in protein lysate buffer (7 M urea, 2 M thiourea, 4% CHAPS, 40 mM DTT, and 0.5% IPG buffer, pH 4–7). The sample was shaken twice for 1 hour at 4°C and sonicated for 15 min to lyse the proteins. The insoluble material was removed by centrifugation at 40 000 × g for 1 hour at 4°C, and the protein concentration was determined using an RC DC Protein Assay Kit I (Bio-Rad). The protein samples were stored at −80°C.

### Two-dimensional electrophoresis

The first dimension was performed using IPG strips (Immobiline DryStrip pH 4–7 NL, 24 cm; GE Healthcare Bio-Sciences). The strips were rehydrated for 16 hours in 450 μl of rehydration solution (7 M urea, 2 M thiourea, 2% CHAPS, 18 mM DTT, 0.5% IPG Buffer 4–7, 0.002% bromophenol blue, and lysate solution containing 600 μg of protein). The IEF was performed in the Ettan IPGphor System (GE Healthcare Bio-Sciences) following the manufacturer’s protocol.

After IEF, the strips were equilibrated in equilibration buffer (50 mM Tris-HCl pH 8.8, 6 M urea, 30% glycerol, 2% SDS, 0.002% bromophenol blue, 1% DTT and 2.5% iodoacetamide) for 15 min each. Then, the strips were placed on 12.5% ExcelGel SDS gel and blocked with blocking buffer (0.5% agarose, 0.002% bromophenol blue in SDS electrophoresis buffer). For the second dimension, the proteins were separated using Ethan DALTsix System (GE Healthcare Bio-Sciences). Electrophoresis was performed at 20°C with 3.5 W/gel for 30 min, followed by 15 W/gel until completion.

### Staining and image analysis

The gels were stained with Coomassie Brilliant Blue G-250. Images were immediately acquired using a Uniscan M1600 (Unisplendour Corporation Limited) to avoid fading. Spot detection and quantification were performed using Image Master 2D Platinum version 6.0 software (GE Healthcare Bio-Sciences). The relative volume (RV) represents the relative abundance of the abundant proteins. The RV was estimated from the actual volume of each protein spot on the gels via homogenization. The relatively stable spots used for determining RV were those present in three gels of each sample. Differentially abundant proteins were the spots with significant differences according to a t-test (P <0.05) by analysing the variance of each spot’s RV.

### Enzyme digestion and protein identification

The enzymatic reaction was performed before MS analysis. The protein spots were cut from the gels with modified pipette tips. To decolorize the spots, 100 μl of 25 mM ammonium bicarbonate/50% acetonitrile was added, and the spots were incubated for 30 min, followed by 50% acetonitrile dehydration for 15 min and 100% acetonitrile dehydration. The spots were digested by trypsin overnight at 37°C, and the digested solution was used directly for MS analysis. For protein spots with low concentrations, 80 μl of 5% trifluoroacetic acid (TFA) was added to the enzymatic solution, which was incubated for 1 hour at 40°C. The supernatant was collected into a new tube. Then, 80 μl 2.5% TFA/50% acetonitrile was added to the old tube for 1 hour at 30°C. The combined extracts were dried by using a vacuum centrifugal evaporator.

Peptide mass fingerprinting (PMF) analysis was performed using m/z MoverZ software. The parameters were set as follows. Mode, m + H; S/N, 4–6 (according to the mass spectrum quality); centroid value, 5; resolution, 4000–6000. Trypsin self-cutting peptides (906.51 Da and 2273.16 Da) were used as internal standards for 2-point calibration. The data from the samples were filtered by Peak Erazor v1.45 software to remove interference from the trypsin self-cutting and keratin peptide peaks. Then, the PMF information was retrieved from the Matrix Science website (http://www.matrixscience.com). The parameters were set as follows. Database, NCBI protein database; peptide mass property, single charge and [M + H] +; classification, Viridiplantae (green plants); mass error range, ± 100 ppm; error cut points, 1.

The amino acid sequences of the protein spots were identified by position-specific iterated and pattern-hit initiated BLAST (PSI-BLAST and PHI-BLAST, respectively) by using the NCBI database (http://www.ncbi.nlm.nih.gov/BLAST/) to obtain the functional domains and functional annotations. To identify the gene ontology (GO) terms, the annotations for each protein were imported into Blast2GO.

## Results

### Culture of male and hermaphroditic gametophytes

**Fig 1:**
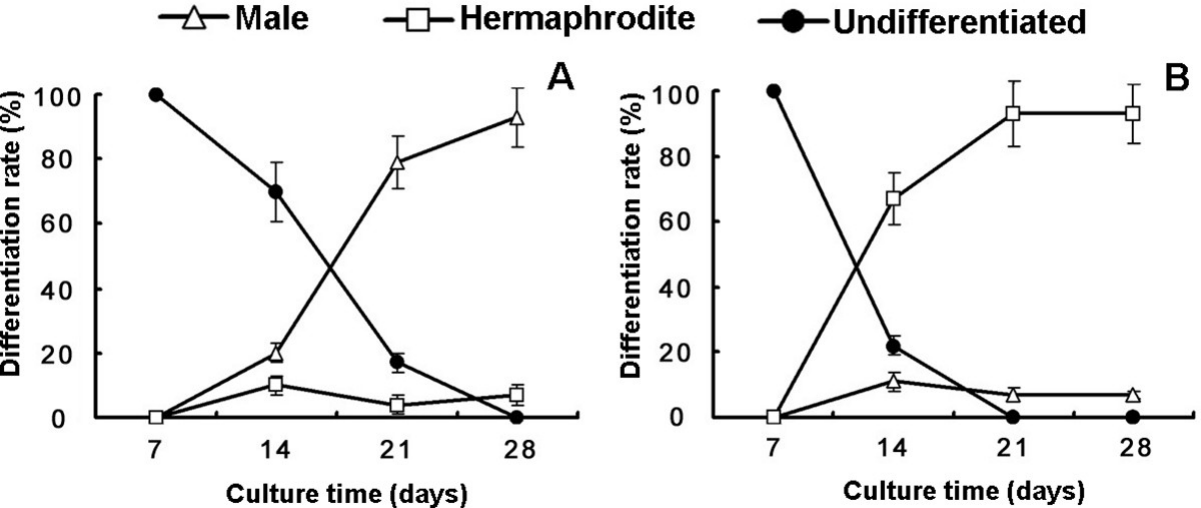
The sex expression of *C*. *thalictroides* gametophytes. (A) Cultures grown on Knop's medium; (B) Cultures grown on MS medium. Three replicates were used for each sample. The bars represent the mean±SD.

**Fig 2:**
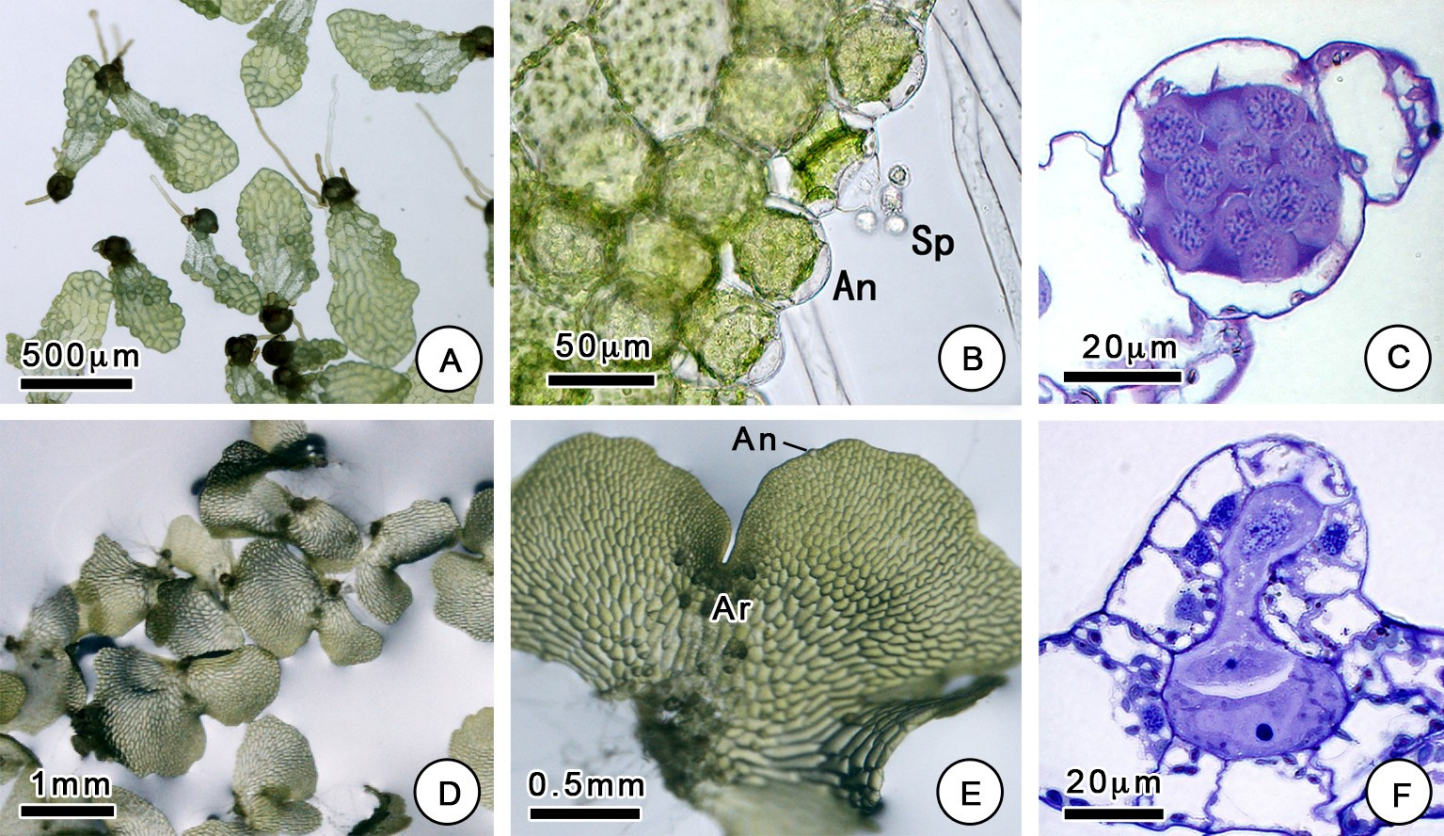
Morphological features of *C*. *thalictroides* gametophytes. (A) Male gametophytes, (B, C) with antheridium (An) and sperm (Sp), observed by microscopy. (D, E) Hermaphroditic gametophytes and (F) archegonium (Ar) observed by microscopy.

Our results suggest that changes in culture media can have a dramatic impact on sex expression (Fig 1). Normally, gametophytes cultured on MS medium begin to differentiate sexually on the 14th day after sowing, and complete sex expression, with the significant development of sexual organs, occurs after approximately 21 days. On MS medium, the spores mainly grew into hermaphroditic gametophytes, which have a heart-like or eccentric shape with a width greater than the length (Fig 2). Hermaphroditic gametophytes have one or several archegonia below the growing point and a few antheridia at the base or margin. However, the sexual differentiation of gametophytes cultured on Knop's medium occurred later, and the gametophytes could be differentiated at approximately 28 days. Unlike the spores grown on MS medium, most of the spores grown on Knop's medium grew into male gametophytes. Male gametophytes show a spoon-like shape with a length that is greater than the width and produce many antheridia at the margin and surface. Male and hermaphroditic gametophytes are different not only in their shape but also in their size (S1 Table). As was the case for the antheridiogen-induced gametophytes, the males were significantly smaller than the hermaphroditic gametophytes [17]. It was obvious that there were more antheridia on the male gametophytes. The proportion of vegetative cells in males was less than that in hermaphrodites. This may be related to the low nutritional requirements of male gametophytes, which will die after sperm release.

### Photochemical efficiency of male and hermaphroditic gametophytes

**Fig 3:**
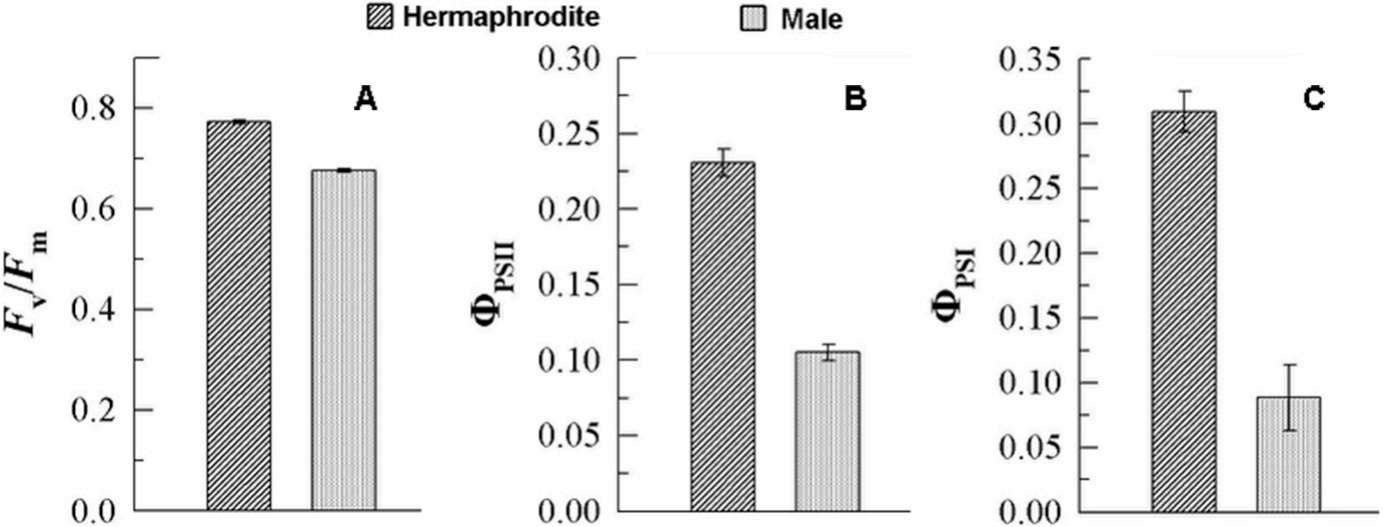
Photosynthetic efficiency of *C*. *thalictroides* gametophytes. (A) The activity of the chlorophyll fluorescence parameter (Fv / Fm); (B) the activity of photosystem Ⅱ (ФPSⅡ); (C) the activity of photosystem Ⅰ (ФPSⅠ). Error bars indicate standard errors of three biological replicates.

The chlorophyll fluorescence in gametophytes was determined for male and hermaphroditic gametophytes (Fig 3). The results showed that the actual photochemical efficiency (ФPSⅡ and ФPSⅠ) and the potential photochemical efficiency (Fv/Fm) of males were lower than those of hermaphrodites. In the moss *Ceratodon purpureus*, females showed greater values during leaf photochemistry measurements than males [23]. The sexual dimorphism was female-biased, which is consistent with our results. Higher photochemical efficiency could provide more energy for the development of archegonia, fertilization, and the growth of sporophytes.

### Protein abundance analysis

**Fig 4:**
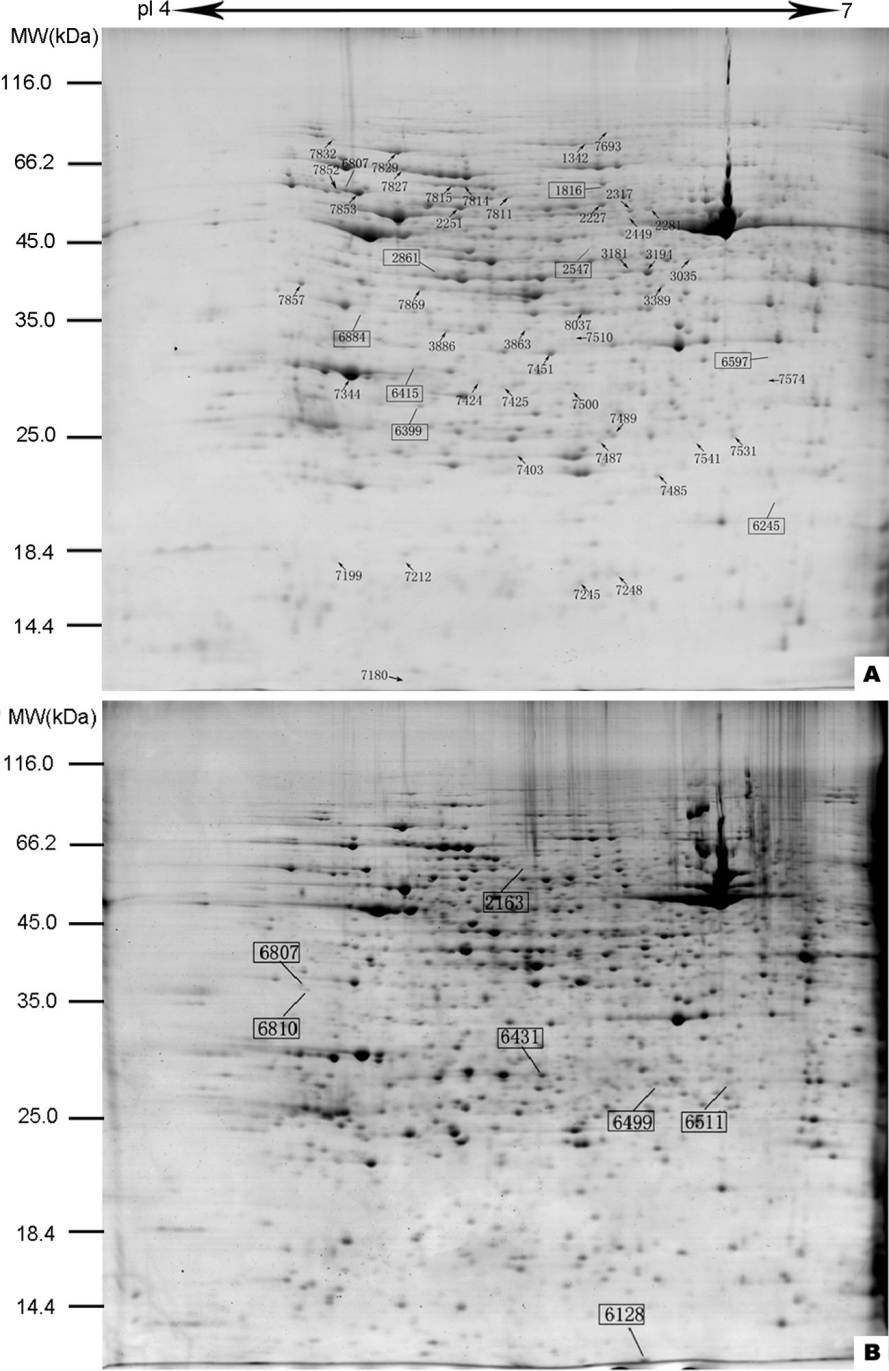
Representative 2-DE images of proteins from *C*. *thalictroides* gametophytes. (A) Protein gel from hermaphroditic gametophytes; (B) protein gel from male gametophytes. Fifty-seven differentially abundant proteins are marked with numbers on the gels. Squares refer to specific proteins. Molecular weight (MW) in kDa and pI of proteins are indicated on the left and top of the gels, respectively. Detailed information can be found in Table 1.

The protein samples were prepared using pH 4–7 IPG strips, separated by 12.5% SDS-PAGE, and stained with Coomassie Brilliant Blue. Then, the gel images were scanned to acquire the protein abundance profiles of the hermaphroditic and male gametophytes (Fig 4). Image analysis revealed the quantitative and qualitative differences in spot intensity in the two types of gametophytes. There were 1136 ± 55 spots observed in hermaphrodites and 1130 ± 65 spots observed in males. Among them, abundance changes of more than 1.5-fold and significant differences according to t-tests were used to identify the differentially abundant protein spots. Quantitatively, there were 57 differentially abundant protein spots, among which 18 protein spots showed higher abundance in male gametophytes and 24 showed higher abundance in hermaphrodites. Seven protein spots were unique to male gametophytes and 8 were unique to hermaphroditic gametophytes.

### Protein identification and functional classification

All 57 protein spots were checked by mass spectrometry, and 33 proteins were identified (Table 1). A good correlation between theoretical and experimental pI was shown, but some differences in MW were observed. For some spots, a higher MW than that reported in the database was observed, possibly due to the absence of mature forms of the proteins or the presence of sequences corresponding to only a fragment of a protein. The identified proteins were mainly involved in protein folding and refolding (33%), photosynthesis and chloroplasts (24%), metabolism (24%), protein synthesis (6%) and cell structure (13%).

**Table 1:** Differentially abundant proteins in hermaphroditic and male gametophytes from *C*. *thalictroides*.

Spot No.^a^	Protein description	Reference species	Accession No.^b^	Exp. MW/pI^c^	Thr. MW/pI^d^	#^e^	SC^f^	Ratio^g^
**Protein folding and refolding (11)**								
2251	Chaperonin 60 subunit beta 2	*Ricinus communis*	XP_002523404.1	59354/5.42	64490/5.65	11	163	2.25±0.17
2163	Chaperonin 60–2	*Physcomitrella patens*	XP_024366438.1	62062/5.67	61757/5.88	4	78	-
7814	Heat shock protein 70	*P*. *patens*	XP_024361882.1	73140/5.44	73140/5.94	9	123	1.47±0.22
7815	Heat shock protein 70	*P*. *patens*	XP_024361882.1	64306/5.39	73140/5.94	7	183	1.68±0.07
7827	Heat shock protein 70	*Oryza sativa*	ACJ54890.1	76511/5.20	71945/5.30	17	423	1.58±0.42
7829	Heat shock protein 90–1	*Nicotiana benthamiana*	AAR12193.1	80339/5.18	80339/4.94	22	431	0.62±0.11
7857	Peptidyl-prolyl cis-trans isomerase	*Spinacia oleracea*	XP_021866579.1	50832/4.84	50068/5.29	5	106	2.01±0.38
7852	Rubisco large subunit-binding protein subunit alpha	*R*. *communis*	XP_002534347.2	62164/4.95	53280/5.25	9	94	1.45±0.13
7853	Rubisco large subunit-binding protein subunit alpha	*Sorghum bicolor*	XP_002440887.1	60913/5.03	60913/5.07	9	121	1.77±0.15
2227	Rubisco large subunit-binding protein subunit beta	*Zea mays*	NP_001306697.1	59583/5.89	61968/5.42	10	289	1.59±0.21
2317	T-complex protein 1 subunit beta	*Vitis vinifera*	XP_002285912.1	58957/6.08	57628/5.60	11	112	0.63±0.07
**Photosynthesis and chloroplast (8)**								
1342	ATP-dependent Clp protease	*Vitis vinifera*	XP_010663794.1	102347/6.32	99169/6.09	19	239	1.63±0.26
7424	Lactoylglutathione lyase	*P*. *patens*	XP_024375349.1	34416/5.49	29627/5.23	5	168	1.99±0.09
3181	LL-diaminopimelate aminotransferase	*P*. *patens*	XP_024392750.1	53048/6.08	50746/8.00	3	113	1.69±0.14
7869	Magnesium-chelatase subunit ChlI	*V*. *vinifera*	RVW29648.1	49864/5.39	46692/5.68	11	108	2.13±0.21
7344	Oxygen-evolving enhancer protein 1	*Populus trichocarpa*	XP_002307234.1	35348/5.01	35348/5.89	6	402	1.70±0.09
7832	Ribulose bisphosphate carboxylase/oxygenase activase	*Populus trichocarpa*	XP_002312110.3	83226/4.94	40277/5.36	10	386	1.92±0.03
2281	Ribulose-1, 5-bisphosphate carboxylase/oxygenase large subunit	*Palhinhaea pendulina*	CAC22277.1	59241/6.15	49006/6.49	12	73	4.28±0.85
2449	Ribulose-1, 5-bisphosphate carboxylase/oxygenase large subunit	*Dryopteris sublacera*	ABF59846.1	58170/6.08	49148/6.54	14	91	0.63±0.01
**Metabolism (8)**								
7811	2,3-bisphosphoglycerate-independent phosphoglycerate mutase	*Mesembryanthemum crystallinum*	Q42908.1	60476/5.61	61316/5.39	4	85	1.53±0.16
3035	3-ketoacyl-CoA thiolase 2	*Prosopis alba*	XP_028773475.1	54155/6.30	48962/6.36	8	168	0.42±0.02
7510	Arginase 1	*P*. *patens*	XP_024376768.1	42118/5.86	37423/6.06	2	76	0.64±0.05
7451	Cysteine synthase-like	*P*. *patens*	XP_024360091.1	38585/5.77	41650 /8.21	6	223	1.62±0.43
3389	Glutamine synthetase	*Cryptomeria japonica*	BBA84049.1	50238/6.20	39732/5.96	3	208	0.53±0.10
7531	Glutathione S-transferase	*V*. *vinifera*	XP_002263395.1	27676/6.47	25740/6.06	2	92	2.07±0.29
7487	Glutathione S-transferase DHAR1	*P*. *patens*	XP_024357492.1	74303/5.96	25502/7.99	4	92	0.53±0.01
8037	Malate dehydrogenase	*Beta vulgaris*	NP_001290006.1	45437/5.90	35810/5.89	7	97	0.55±0.06
**Protein synthesis (2)**								
3194	Elongation factor tub	*N*. *sylvestris*	XP_009772722.1	52769/6.15	52769/5.95	6	152	1.72±0.07
7245	Translation initiation factor 5A	*Dendrocalamus sinicus*	ABW78939.1	15509/5.89	17752/7.08	5	187	0.63±0.02
**Cell structure (4)**								
2861	Actin 2	*Anemia phyllitidis*	AAC64127.1	53023/5.33	41827/5.31	15	340	+
6415	Actin	*P*. *patens*	XP_024374057.1	36460/5.25	41810/5.3	6	76	+
1816	Actin	*Mesostigma viride*	O65316.1	69752/5.97	41790/5.3	6	84	+
7199	Centrin	*Pterosperma cristatum*	CAA58719.1	17020/4.94	15320/4.38	3	179	0.25±0.06

^a^: the numbering corresponds to the matched IDs in the 2D gels

^b^: the database accession number from the NCBI protein database

^c, d^: the experimental and theoretical molecular weight (Da) and pI of the identified proteins

^e^: number of peptides identified by MS/MS

^f^: mascot score resulting from the LC-MS/MS search

^g^: the relative fold change in abundance levels when compared with the abundance in male gametophytes (p ≤ 0.01); (+) denotes a unique spot in hermaphroditic gametophytes, (-) denotes a unique spot in male gametophytes. The average and standard deviation are presented.

## Discussion

Photosynthesis plays an important role in plant growth and development, and our results show that hermaphroditic gametophytes have higher photochemical efficiency than males. Ribulose-1,5-bisphosphate carboxylase/oxygenase (rubisco), oxygen-evolving enhancer protein 1 (OEE), and magnesium-chelatase are considered to be directly involved in photosynthesis. Their abundance is positively correlated with photosynthesis [24, 25]. Most forms of rubisco have been previously described as accumulating in females of the fern *Blechnum spicant* [22]. Our results also indicate that rubisco, OEE, and magnesium-chelatase were enriched in hermaphrodites in *C*. *thalictroides* (Table 1). Chaperones and proteases ensure correct protein folding and prevent the formation of toxic aggregates. Numerous studies have shown that some chaperones and proteases are essential for maintaining photosynthesis stability [26–28]. The rubisco large subunit-binding protein is abundant in plastids, which are essential in photosystem II [29]. All three rubisco large subunit-binding proteins identified in the present investigation showed higher abundance in hermaphrodites. Peptidyl-prolyl cis-trans isomerase (PPIase) is considered to participate in the photosynthetic electron transport chain in the thylakoid membrane [30, 31]. Valledor et al. found that the PPIase level was relatively high in female gametophytes in *B*. *spicant* [22]. In line with these results, we found that the abundance level of PPIase in hermaphroditic gametophytes of *C*. *thalictroides* was approximately twice that found in males. We propose that a high level of PPIase activity is necessary for female or hermaphroditic development in ferns. Members of the HSP70 family are highly represented in hermaphroditic gametophytes. In *Chlamydomonas reinhardtii*, HSP70 has been shown to play a role in the protection of photosystem II against damage in photoinhibitory conditions [32, 33]. This implies that HSP70 may be involved in protecting against damage caused by photosynthesis in hermaphrodites of *C*. *thalictroides*.

Microscopic observations showed that there were more chloroplasts in the hermaphroditic gametophytes, so some of the proteins localized to chloroplasts were abundant more highly in the hermaphroditic gametophytes. These proteins are involved in maintaining the stability of the chloroplast and allowing higher photosynthetic utilization in the hermaphroditic gametophyte. It was found that lactoylglutathione lyase regulates plant adaptation to various abiotic and biotic stresses by improving methylglyoxal detoxification and reducing oxidative damage, thereby measuring the improved protection of chloroplast and mitochondrial ultrastructure and the maintenance of photosynthetic efficiency under stress conditions [34]. The ATP-dependent Clp protease maintains protein homeostasis in plastids. Tobacco lines with knockdown of Clp protease showed pigment deficiency, alterations in leaf development, leaf variegations, and impaired photosynthesis [35]. LL-diaminopimelate aminotransferase (LL-DAPAT) is a key gene involved in the synthesis of lysine. Mutations in the LL-DAPAT gene lead to reduced photosynthesis and impaired plant growth [36].

Male gametophytes grow under relatively poor conditions, so some proteins with defence characteristics are relatively highly abundant in them. For example, dehydroascorbate reductase (DHAR), malate dehydrogenase, and arginase 1 were abundant in male gametophytes. DHAR, a type of glutathione S-transferase, can catalyse the synthesis of ascorbic acid by dehydroascorbate, which is a cofactor in the xanthophyll cycle and a highly effective antioxidant that helps plants excrete reactive oxygen [37]. Malate dehydrogenase might provide building material and energy for the biosynthesis of defence compounds [38]. Arginase 1 has been shown to mobilize nitrogen storage as well as fine-tune development and defence mechanisms against stress [39]. HSP90 is necessary for proper defence signal transduction via the stabilization of resistance proteins [40, 41]. In *Arabidopsis*, the abundance of HSP90 increased significantly in conditions with high heat, low temperatures, high salt, or heavy metals [42–44]. As has been observed in *B*. *spicant* [22], HSP90 may be associated with male development.

In addition, we observed during culture that male gametophytes would gradually die after their antheridia matured. According to our results, some proteins involved in leaf senescence, such as 3-ketoacyl-CoA thiolase 2 (KAT2), appear to be distributed in male gametophytes. KAT2 has been reported to be responsible for the majority of jasmonic acid biosynthesis [45, 46]. In *Arabidopsis*, KAT2 was confirmed as an essential component for the timely onset of leaf senescence [47]. The increase of KAT2 in males may result in senescence in male gametophytes that occurs earlier than we observed.

The obvious difference in sexual organs is the main feature that allows for distinguishing between male and female gametophytes. The differential abundance of cytoskeletal proteins detected in this study may be involved in the formation of sexual organs. Centrin, a member of a family of calcium-binding phosphoproteins, is distributed in the centrosomes or surrounding matrix in eukaryotes [48, 49]. In green algae, it was identified as a major component of the basal body associated with contractile striated flagellar roots [50]. In ferns, centrin is present in or near the blepharoplast and the multilayered structure of spermatids, which are necessary for the formation of the motile apparatus in spermatids of *Marsilea* [51]. In this study, the abundance of centrin in male gametophytes was found to be very high, approximately four times greater than that found in hermaphrodites. This result indicates that centrin is primarily involved in male gametophyte development and may be involved in the sperm formation process. In our study, three actin proteins were abundant specifically in hermaphroditic gametophytes. Actin is a family of globular multifunctional proteins that form microfilaments. Eukaryotic actin is required for numerous cellular processes, including the maintenance of cell shape, cell development and movement, gene expression, signal transduction, and responses to biotic and abiotic stress [52]. A large amount of actin has been found to be synthesized during spore germination of *Equisetum arvense* [19]. We speculate that the actin proteins detected in hermaphrodites are related to the structure of the archegonia. The differences in the abundance levels of cell structure proteins revealed the presence of large differences in cytoskeletal dynamics in sex expression in gametophytes.

## Conclusions

Overall, we generated two forms of gametophytes with sex expression differences using different culture methods. The photochemical efficiency in male gametophytes was lower than that in hermaphroditic gametophytes, and the levels of proteins involved in photosynthesis were also decreased. In addition, some metabolic proteins had higher activity in male gametophytes, which allowed them to maintain their development under relatively poor nutritional conditions. In addition, we detected several proteins that may be involved in sexual organ formation, such as centrin and actin, and these cytoskeletal proteins may be directly involved in sex expression. These differentially abundant proteins are important for maintaining the different developmental characteristics of hermaphroditic and male gametophytes in *C*. *thalictroides*.

## Supporting information

S1 Table Gametophyte size and cell number of *C*. *thalictroides*. (XLSX)
